# Physical activity, fitness, and cardiac autonomic function among adults born postterm

**DOI:** 10.1093/aje/kwae150

**Published:** 2024-06-24

**Authors:** Päivi Oksanen, Marjaana Tikanmäki, Mikko P Tulppo, Maisa Niemelä, Raija Korpelainen, Eero Kajantie

**Affiliations:** Research Unit of Clinical Medicine, University of Oulu, Oulu, Finland; Population Health Unit, Finnish Institute for Health and Welfare, Helsinki and Oulu, Finland; Medical Research Center Oulu, Oulu University Hospital and University of Oulu, Oulu, Finland; Research Unit of Clinical Medicine, University of Oulu, Oulu, Finland; Population Health Unit, Finnish Institute for Health and Welfare, Helsinki and Oulu, Finland; Medical Research Center Oulu, Oulu University Hospital and University of Oulu, Oulu, Finland; Medical Research Center Oulu, Oulu University Hospital and University of Oulu, Oulu, Finland; Research Unit of Biomedicine and Internal Medicine, University of Oulu, Oulu, Finland; Medical Research Center Oulu, Oulu University Hospital and University of Oulu, Oulu, Finland; Research Unit of Health Sciences and Technology, University of Oulu, Oulu, Finland; Medical Research Center Oulu, Oulu University Hospital and University of Oulu, Oulu, Finland; Research Unit of Population Health, University of Oulu, Oulu, Finland; Department of Sports and Exercise Medicine, Oulu Deaconess Institute Foundation sr., Oulu, Finland; Research Unit of Clinical Medicine, University of Oulu, Oulu, Finland; Population Health Unit, Finnish Institute for Health and Welfare, Helsinki and Oulu, Finland; Medical Research Center Oulu, Oulu University Hospital and University of Oulu, Oulu, Finland; Department of Clinical and Molecular Medicine, Norwegian University of Science and Technology, Trondheim, Norway

**Keywords:** cardiac autonomic function, cardiorespiratory fitness, heart rate recovery, physical activity, postterm birth

## Abstract

Recent studies have suggested that adverse outcomes of postterm birth (≥42 completed weeks of gestation), including increased cardiometabolic risk factors, impaired glucose metabolism, and obesity, may extend into adulthood. We studied interconnected determinants of cardiovascular health, including physical activity (PA; based on accelerometry for 2 weeks), muscular strength (measured by handgrip strength), cardiorespiratory fitness (CRF; measured by 4-min step test), and cardiac autonomic function (heart rate [HR] recovery, HR variability, and baroreflex sensitivity) among 46-year-old adults from the Northern Finland Birth Cohort born postterm (*n* = 805) and at term (*n* = 2645). Adults born postterm undertook vigorous PA 2.0 min day^−1^ (95% CI, 0.4-3.7) less than term-born adults when adjusted for sex, age, and maternal- and pregnancy-related covariates in multiple linear regression. Postterm birth was associated with reduced CRF, based on a higher peak HR (2.1 bpm; 95% CI, 0.9-3.4) and slower HR recovery 30 s after the step test (−0.7 bpm; 95% CI, −1.3 to −0.1). Postterm birth was associated with less PA of vigorous intensity and CRF and slower HR recovery in middle age. Our findings reinforce previous suggestions that postterm birth should be included as a perinatal risk factor for adult cardiometabolic disease.

## Introduction

The rates of postterm births (≥42 completed weeks of gestation) have varied widely over the decades depending on the method of measuring gestational age and the obstetric practice of inducing delivery. In Europe and the United States, the rates of postterm births have varied from 20% (observed in 1960s) to more recent rates at approximately 0.4% of births.^1^^‑^^3^ A recently published study^4^ from the United States revealed a decline in postterm birth rates from 0.43% to 0.27% during 2014 to 2022.

Postterm pregnancy is associated with a higher risk of stillbirth and neonatal and postneonatal death,^5^^,^^6^ labor dysfunction, placental problems, and macrosomia (which may lead to fetal distress, the need for cesarean section, obstetric trauma, a need for neonatal intensive care, and problems with breastfeeding).^5^^,^^7^^,^^8^ Recent studies have suggested that the adverse outcomes of postterm birth may extend into childhood and adulthood. Compared with term birth, the postterm birth is associated with higher rates of overweight in adults,^1^^,^^9^ reduced exercise capacity in adolescents,^10^ and impaired glucose metabolism in children.^11^ However, most of these studies were small and involved some uncertainty.

Physical activity (PA), muscular strength, and cardiorespiratory fitness (CRF) are important determinants of cardiovascular health.^12^^,^^13^ Physical activity and CRF are negatively associated with all-cause mortality^14^^‑^^17^ and positively associated with healthy cardiac autonomic regulation.^18^^‑^^21^ Moderate- to vigorous-intensity PA is associated with lower rates of death for cardiovascular health–related reasons. When solely accounting for vigorous-intensity PA (VPA), the association seems to be stronger when compared with moderate-intensity PA (MPA).^22^^,^^23^ Low muscular strength at midlife predicts disability at old age, as well as all-cause and cardiovascular mortality.^24^^,^^25^

Healthy cardiac autonomic function is reflected in a lower heart rate (HR) at rest, higher HR variability and baroreflex sensitivity, and faster HR recovery.^20^^,^^26^^‑^^28^ Impaired cardiac autonomic function is associated with all-cause and cardiovascular mortality.^29^^,^^30^ Long-term blood pressure variability is associated with risk for cardiovascular disease and death.^31^

We investigated PA, CRF, muscular strength, and cardiac autonomic function in adults born postterm, and evaluated the contribution of PA and body mass index (BMI) to CRF and cardiac autonomic function. Based on previously observed higher rates of overweight^1^^,^^9^ and reduced exercise capacity among individuals born postterm, we hypothesized that adults born postterm would undertake less PA and have lower muscular strength, CRF, and cardiac parasympathetic activity at 46 years of age compared with term-born adults. Moreover, we hypothesized that PA and BMI would mediate the association between postterm birth and cardiac autonomic function.

## Methods

Information on health, lifestyle, and socioeconomic status was collected from the Northern Finland Birth Cohort 1966 (NFBC1966),^32^ a prospective cohort study including all children to mothers living in northern Finland with an expected date of birth during 1966 (96.3% of all 1966 births; *n* = 12 058 live births). The design and data collected in the NFBC1966 study have been described in detail.^32^^,^^33^

Since spring 2012, cohort members have been invited to clinical examinations when they are age 46 years. Postal surveys were conducted prior to the clinical examinations. Of the 10 321 invited individuals, 66% (*n* = 6825) participated in postal surveys, and 56.5% (*n* = 5832) participated in clinical examinations. The protocol for the 46-year visit has been described in detail.^19^

On the clinical examination day, height, weight, and Homeostatic Model Assessment for Insulin Resistance (HOMA-IR) were measured. A threshold of 1.9 for HOMA-IR was applied based on a Finnish study.^34^ Additionally, CRF, muscular strength, and cardiac autonomic function were measured, and 2-week PA monitoring initiated. Based on the health questionnaire in the postal surveys, the participants were classified as current smokers or nonsmokers (including former smokers), and as at-risk alcohol consumers when the reported daily alcohol use was more than 40 g of pure ethanol for men and 20 g of pure ethanol for women.

All participants were provided written informed consent. Ethics approvals for NFBC1966 were granted by the Regional Medical Research Ethics Committee of North Ostrobothnia Hospital District, Finland (EETTMK no. 3/97, 17/2003 ja 94/2011). The study was conducted in accordance with the Declaration of Helsinki.

### Exposure, and inclusion and exclusion criteria

**Figure 1:**
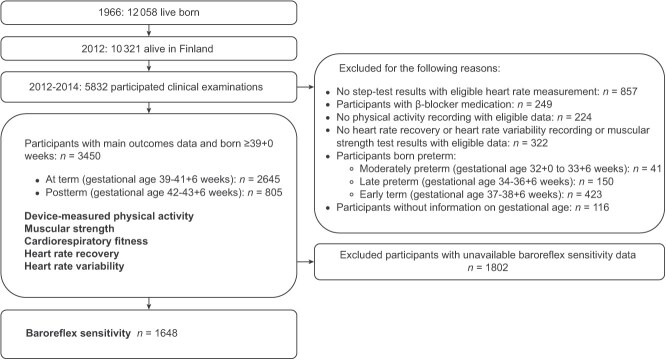
The selection of the study population from the Northern Finland Birth Cohort 1966.

Our exposure was postterm birth, defined as gestational age ≥ 42 completed weeks. Of 805 postterm participants, 97% (*n* = 780) were born at 42-43 + 6 weeks, and 3% (*n =* 25) were born at ≥44 + 0 weeks. Our comparison group (ie, control participants) was those born at term, during 39 to 41 completed weeks of gestation (*n* = 2645). Gestational age was based on the last menstrual period reported by the mother. We included only participants born during those weeks 39-44 + 0 and who had HR and PA data completed. Baroreflex sensitivity data were available only for 48% of the participants (*n* = 368 postterm; *n* = 1280 term). Exclusions and the number of participants included in the analyses are described in Figure 1.

### Perinatal and childhood data

Birth weight SD values were determined according to Finnish standards. Small for gestational age was defined as a birth weight of less than −2 SD below the mean for gestational age, and large for gestational age as a birth weight of > 2 SD above the mean for gestational age, both adjusted for sex.^35^ Maternal-, pregnancy-, and childhood-related variables (Table S1) were originally collected from antenatal and child welfare clinics and school health care services. Information about the father’s smoking was asked from the cohort participant or their parents when the cohort participant was 14 years old.^32^

### Attrition analyses

**Figure 2:**
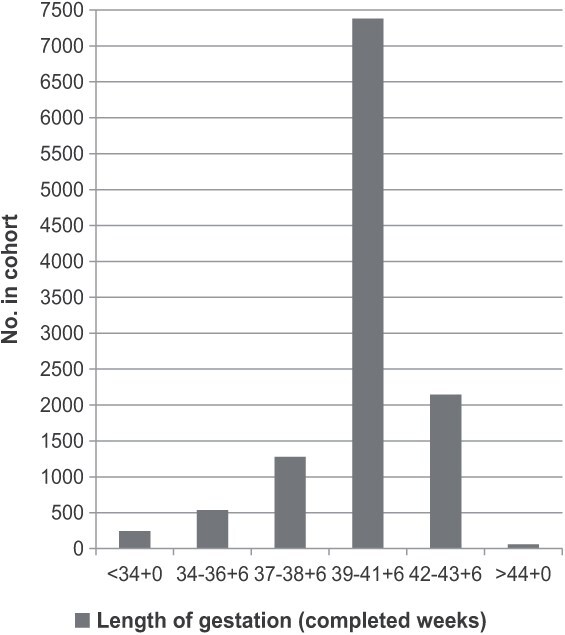
Distribution of study population across the gestational age among 46-year-old adults from Northern Finland Birth Cohort 1966.

A detailed NFBC1966 nonparticipant analysis has been published.^32^^,^^33^ Gestational age values were available for 11 640 NFBC1966 cohort members (Figure 2). In the present study, only postterm and term-born adults were included. Comparisons of participants and cohort members who had insufficient data are shown in Table S2. Mothers of participants were less likely to smoke, more likely to be of normal weight, and more likely to have a professional socioeconomic position. Participants were also more likely to have been members of a sports club in adolescence, although this was statistically significant only among the term-born group.

### PA and muscular strength

Device-measured daily sedentary behavior and PA were determined using a waterproof, wrist-worn, accelerometer-based Polar Active monitor (Polar Electro). The Polar Active device calculates energy expenditure as metabolic equivalents of task (MET) every 30 s based on the acceleration sensed. Only data from waking hours were analyzed. The user’s height, weight, age, and sex were used as background information. The active monitor was blinded and unprovided any feedback to the user.

Participants were instructed to wear a monitor on the nondominant hand for 24 hours (except in the sauna bath) for at least 14 days.^36^ The first day when activity monitors were given was excluded from the analysis. An eligible day was at least 600 min day^−1^ wear time during waking hours.^37^ Participants with at least 4 valid measurement days were included in the analyses. Daily averages (min day^−1^) of 4 activity levels were calculated: sedentary behavior, light PA (LPA), MPA, and VPA.^38^

Sedentary behavior (including reclining, sitting, standing) was assessed as all PA with an intensity of 1-2 MET, LPA as all PA with an intensity of 2-3.5 MET, MPA as all PA with an intensity of 3.5-5 MET, and VPA as all PA with an intensity of at least 5 MET.^39^ These intensity-level thresholds provided by the device manufacturer yielded more comparable results for sedentary time and moderate PA than traditionally used cutoff values.^40^ Polar Active has shown close agreement with the double-labeled water technique in assessing energy expenditure during exercise and daily life.^36^^,^^41^

For self-reported frequency level of PA (gathered from a multi-item questionnaire mostly at a different time from the device-measured PA), the participants were asked how often during their leisure time they participated in PA that causes at least some sweating and getting out of breath, corresponding to moderate- to vigorous-intensity PA. Frequency of self-reported PA was classified as high PA (4-7 times a week), moderate PA (1-3 times a week), and low PA (less than once a week).

Muscular strength was determined by the maximal handgrip strength test^12^^,^^42^ of the dominant hand (hand dynamometer; Newtest). The highest of 3 consecutive measurements was included in the analyses.

### CRF and HR recovery

Cardiorespiratory fitness was estimated based on peak HR (HRpeak; in bpm) during a submaximal 4-min step test,^19^^,^^43^^‑^^45^ and heart rate recovery (HRR) was determined after the step test. Peak HR and HRR were measured using a Polar HR monitor (RX800RC; Polar Electro). High HR and slow HRR indicate low CRF. The HRR at 30 s after the step test (HRR30), HRR 60 s after the step test (HRR60), and steepness of the decrease of HR during 30 s after the step test (HRRslope; the steepest 30-s slope, was measured in beats s^−1^) were measured to study the rate and angle of decrease in HR after the step test. All HRR values were normalized according to HRpeak.

### HR variability and baroreflex sensitivity during an orthostatic test

Heart rate variability (HRV) during an orthostatic test in 3-min seated and 3-min standing phases was measured to describe cardiac sympathovagal activity (Polar HR monitor RX800RC; Polar Electro). The R-R interval (RRi; measured in milliseconds)—the time elapsed between 2 successive R-waves of the QRS signal—was computed for further analyses. The RRi and the root mean square of successive differences between normal heartbeats were analyzed to describe cardiac vagal activity.^46^^,^^47^ The short-term scaling exponent of RRi detrended fluctuation analysis was analyzed to estimate the cardiac sympathovagal balance^48^ and detect slight changes in the RRi dynamics.^49^ Low-frequency power (0.04-0.15 Hz, given as ln ms^2^) and high-frequency power (0.15-0.40 Hz, also given as ln ms^2^) of HRV, and the ratio of low-frequency power to high-frequency power were calculated to express sympathovagal changes in cardiac autonomic regulation.^47^ There were ≥80% eligible data for both phases (seated and standing) and these were included in analyses.^19^

Baroreflex sensitivity was measured during the orthostatic test to describe the capability of the autonomic nervous system to respond to increased blood pressure by increasing vagal and decreasing sympathetic activity in cardiac autonomic regulation, and to detect possible autonomic dysfunction.^30^^,^^50^ Diastolic and systolic blood pressure (mm Hg), and low-frequency systolic blood pressure variability (measured in ms^2^)^46^ and the low-frequency band as the square root of the ratio of RRi to systolic blood pressure spectral component ratio (ms to mm Hg) to describe possible higher sympathetic activity, were measured in seated and standing positions using various instruments: Cardiolife (Nihon Kohden), MLT415/D Nasal Temperature Probe (ADInstruments), and Nexfin (BMEYE Medical Systems).^19^^,^^20^

### Statistical analyses

The data were analyzed using IBM SPSS Statistics, version 27.0 for Windows. The level of 2-sided statistical significance was set at *P* < .05 with 95% CIs. Throughout the article, CIs marked with an asterisk are significant at *P* < .05. Differences in characteristics between study groups and between participants and nonparticipants were assessed using the Student *t* test for continuous variables and Pearson χ^2^ test for categorical variables. Group differences in the main outcomes were evaluated by linear regression.

A multiple linear regression analysis (input variables were entered simultaneously) was performed to determine the contributions of the covariates (Table S1) on outcomes. Model 1 included sex and age at assessment. Model 2 included the model 1 variables plus maternal-, pregnancy-, and childhood-related factors as additional confounders. Model 3 included model 2 variables plus potential adulthood lifestyle and body size–related mediating factors on the causal pathway between postterm birth and the outcomes. Categorical covariates were entered as dummy variables (due to their non-numeric nature), including a separate dummy variable for missing values (Appendix S1, Table S1).

Mediation analysis (PROCESSModel 4 for PROCESS, version 4.1^51^^,^^52^) was conducted to estimate the potential effect of postterm birth on outcomes, and the proportion of the postterm association mediated through PA and BMI. The unstandardized regression coefficients (β) of effects of postterm birth on outcomes were calculated. Mediation analyses were used regarding outcomes with statistically significant group differences in the regression analyses. Using 10 000 bootstrap samples, direct and indirect effect estimates (95% bootstrap CI) of MPA, VPA, and BMI were analyzed to investigate the association between postterm birth and outcomes. The mediation analyses were adjusted for age at assessment and sex.

With a power of 0.8 and an α value of .05, 2-way comparisons allowed us to detect a 0.11 SD difference in any continuous variable between the postterm group (*n* = 805) and the term-born group (*n* = 2645). For a power of 0.90 and an α of .01, the corresponding detectable difference is 0.16 SD. We report standardized detectable differences in SD units as a broad indicator of the study’s ability to detect effects across the range of continuous outcomes examined. The actual minimum detectable difference obviously varies across specific outcomes according to the SD and overall variability of each outcome measure. The standardized 0.11 SD difference corresponds approximately to 2 minutes in device-measured daily VPA, 1.3 kg handgrip strength or 1.7 bpm of maximum HR, meaning that a clinically significant difference can be detected or excluded in this sample size. Sensitivity analysis was performed excluding individuals whose gestational age was 45-46 weeks.

## Results

**Figure 3:**
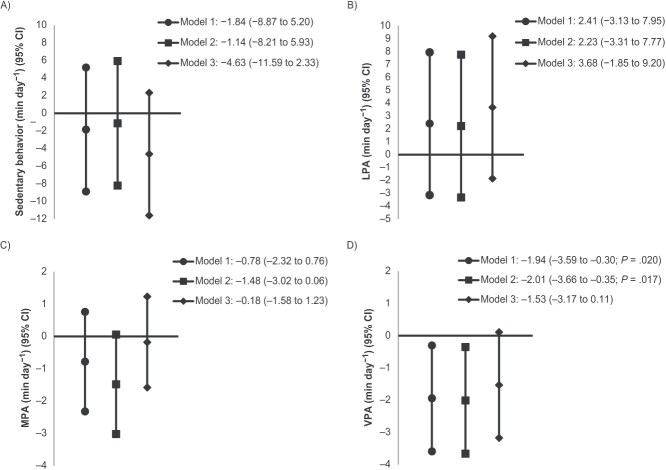
Mean differences in sedentary behavior and physical activity among 46-year-old adults born at term and postterm from Northern Finland Birth Cohort 1966. Mean differences in device-measured sedentary behavior (min day^−1^) (A), light-intensity physical activity (LPA; min day^−1^) (B), moderate-intensity physical activity (MPA; min day^−1^) (C), and vigorous-intensity physical activity (VPA; min day^−1^) (D) in adults born postterm (*n* = 805) compared with term-born control participants (zero line) (*n* = 2645) analyzed by multiple linear regression (95% CIs indicated by error bars). Sedentary behavior, LPA, MPA, and VPA were measured by a wrist-worn accelerometer-based Polar Active monitor. Sedentary behavior was assessed as all activity with an intensity of 1-2 metabolic equivalents of task (MET), LPA as all physical activity with an intensity of 2-3.5 MET, MPA as all activity with an intensity of 3.5-5 MET, and VPA as all activity with an intensity of at least 5 MET. Analyses were adjusted for 3 models, as described in the text. *P* values indicate 2-sided statistical significance for differences between values compared with adults born at term.

**Figure 4:**
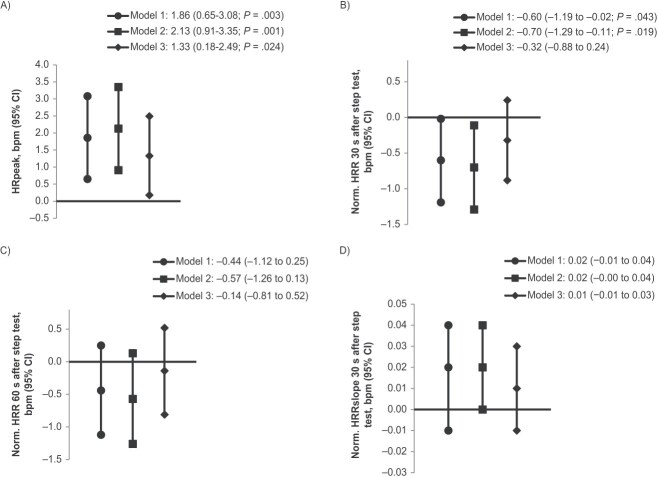
Mean differences in heart rate (HR) and HR recovery (HRR) among 46-year-old adults born term and postterm from Northern Finland Birth Cohort 1966. Mean differences in peak HR (HRpeak; bpm) during a submaximal step test (A), HRR 30 and 60 s (HRR30 and HRR60, respectively, measured in bpm) after the step test (B and C), and the steepest 30-s slope (HRRslope; beats s^−1^) after the step test (D) in adults born postterm compared with term-born control participants (zero line) analyzed by multiple linear regression (95% CIs indicated by error bars). The HRR values were normalized (norm.) according to HRpeak. Analyses were adjusted for 3 models, as indicated in the text. *P* values indicate 2-sided statistical significance for differences between values compared with adults born at term.

The characteristics and outcomes of the study groups are displayed in Tables 1 and 2. The adjusted mean differences obtained from linear regression are presented in Table 2 and in Figures 3 and 4.

**Table 1:** Perinatal (mother and child), neonatal, and childhood characteristics among adults born postterm and at term from the Northern Finland Birth Cohort 1966

**Variable**	**Postterm** ^**a**^ **(*n* = 805)**			**Term** ^**b**^ **(*n* = 2645)**			**Mean difference (95% CI) between groups**	***P*** ^**c**^
	**Mean (SD) or median [IQR]**	**No. (%)**	**No. with missing data**	**Mean (SD) or median [IQR]**	**No. (%)**	**No. with missing data**		
Male sex		385 (47.8)	0		1189 (45.0)	0		.152
Peri- and neonatal characteristic								
Maternal age at delivery	27.4 (6.7)		0	27.8 (6.9)		0	−0.3 (−0.9 to 0.2)	.220
Maternal smoking^d^		94 (11.7)			309 (11.7)			.997
Maternal diabetes^e^		2 (0.2)			0 (0.0)			.052^*^
Maternal hypertension^f^		109 (13.5)			326 (12.3)			.363
Maternal preeclampsia^g^		22 (2.7)			66 (2.5)			.708
Maternal BMI^h^ (kg m^−2^)	23.0 (3.1)		62	23.1 (3.1)		174	−0.1 (−0.3 to 0.2)	.654
BMI < 25.0		595 (73.9)			1,949 (73.7)			
BMI 25.0-29.9		122 (15.2)			442 (16.7)			
BMI ≥ 30.0		26 (3.2)			80 (3.0)			
Multiple deliveries		3 (0.4)			33 (1.2)			.044^*^
Parity	2.80 [3.00]		0	2.86 [3.00]		0	−0.06 (−0.23 to 0.11)	.373^**^
Gestational age, weeks	42.42 (0.77)		5	40.12 (0.75)		0	2.30 (2.25-2.36)	<.001
Birth weight, kg	3.69 (0.47)		0	3.51 (0.47)		0	0.18 (0.15-0.22)	<.001
Birth weight SD value	0.11 (0.98)		5	−0.14 (1.00)		0	0.25 (0.17-0.33)	<.001
Small for gestational age		15 (1.9)			64 (2.4)			.356
Large for gestational age		24 (3.0)			59 (2.2)			.224
Maternal occupation								.616
Professional		126 (15.7)			387 (14.6)			
Manual worker^i^		243 (30.2)			776 (29.3)			
No occupation or not known		436 (54.2)			1482 (56.0)			
Paternal occupation								.509
Professional		212 (26.3)			696 (26.3)			
Manual worker^i^		411 (51.1)			1301 (49.2)			
No occupation or not known		182 (22.6)			648 (24.5)			
Information collected from participant at ~14 years old								
Paternal smoking		225 (28.0)			923 (34.9)			<.001
Membership in a sports club		290 (37.7)			974 (38.3)			.756

Abbreviation: BMI, body mass index.

c Postterm group: those born at ≥42 + 0 weeks of gestation.

^b^ Term group (control participants): those born at weeks 39 + 0 to 41 + 6.

^c^ The *P* values indicate 2-sided statistical significance for differences between the postterm and term groups using Pearson χ^2^ test [no. (%)], Fisher exact test^*^ [no. (%)], Student *t* test [mean (SD)], or Mann-Whitney U test^**^ [median (IQR)].

^d^ Maternal smoking in second month of pregnancy.

^e^ Diabetes mellitus or prediabetes.

^f^ Gestational or chronic hypertension.

^g^ Preeclampsia or superimposed preeclampsia.

^h^ BMI in early pregnancy classified as healthy weight range (BMI < 25.0 kg m^−2^), overweight (BMI 25.0 to 29.9 kg m^−2^), or obesity (BMI ≥ 30.0 kg m^−2^).

^i^ Skilled or unskilled manual worker.

**Table 2:** Measures at 46 years of age among adults born postterm and at term from the Northern Finland Birth Cohort 1966

**Variable**	**Postterm** ^**a**^ **(*n* = 805)**			**Term** ^**b**^ **(*n* = 2645)**			**Mean difference (95% CI) between groups**	***P*** ^**c**^
	**Mean (SD)**	**No., %**	**Missing data (no.)**	**Mean (SD)**	**No., %**	**Missing data (no.)**		
Age, years	46.6 (0.6)			46.6 (0.6)		0	−0.1 (−0.1 to −0.0)	.026
Height, cm	171.2 (9.3)			171.2 (9.1)			0.00 (−0.7 to 0.7)	.986
Weight, kg	77.8 (14.7)			76.1 (14.9)			1.8 (0.6 to 2.9)	.003
BMI (kg m^−2^)^d^	26.46 (4.06)			25.84 (3.92)			0.62 (0.31 to 0.93)	<.001
BMI classified								.002
< 18.5		6 (0.7)			21 (0.8)			
18.5-24.9		306 (38.0)			1,197 (45.3)			
25.0-29.9		345 (42.9)			1,047 (39.6)			
30.0-34.9		122 (15.2)			321 (12.1)			
≥ 35.0		26 (3.2)			59 (2.2)			
Waist to height ratio	0.45 (0.07)		0	0.44 (0.07)		0	0.01 (0.00-0.01)	<.001
HOMA-IR	2.59 (5.30)		26	2.11 (1.69)		76	0.48 (0.10-0.85)	.014
HOMA-IR >1.9^e^		369 (47.4)	26	1,084 (42.2)		76		.011
Current smoker		145 (18.0)			460 (17.4)			.685
Alcohol use (g day^−1^)	10.76 (16.44)		25	1,0.22 (15.23)		106	0.53 (−0.71 to 1.78)	.400
Risky alcohol use^f^		63 (7.8)			191 (7.2)			.565
Device-measured PA^g^ (min day^−1^)								
Sedentary behavior		629.4 (90.2)			628.3 (89.2)		1.0 (−5.8 to 7.8)	.767
Light-intensity PA		281.1 (72.4)			280.5 (70.3)		0.6 (−4.8 to 6.0)	.825
Moderate-intensity PA		37.8 (22.3)			38.2 (22.2)		0.3 (−2.0 to 1.4)	.709
Vigorous-intensity PA		31.7 (20.0)			33.8 (21.0)		2.1 (−3.7 to −0.5)	.009
Self-reported PA^h^			153			491		.534
High		114 (17.5)			358 (16.6)			
Moderate		395 (60.6)			1,279 (59.4)			
Low		143 (21.9)			517 (24.0)			
Dominant-hand grip strength, kg		36.7 (12.2)	9		36.1 (12.4)	40	0.6 (−0.4 to 1.5)	.229
CRF based on HRpeak	149.1 (15.3)			147.4 (15.6)			1.7 (0.4-2.9)	.008
CRF^i^								
High		212 (26.3)			862 (33.1)			.001
Moderate		313 (38.9)			956 (36.1)			.158
Low		280 (34.8)			827 (31.3)			.061
HRR								
HRR30, bpm	17.0 (7.3)			17.6 (7.6)			−0.6 (−1.2 to −0.0)	.046
HRR60, bpm	28.2 (8.7)		5	28.6 (8.7)		22	−0.4 (−1.1 to 0.3)	.257
HRRslope, beats s^−1^	−0.70 (0.27)		4	−0.72 (0.27)		15	0.01 (−0.01 to 0.04)	.176

Abbreviations: BMI, body mass index; CRF, cardiorespiratory fitness; HOMA-IR, Homeostatic Model Assessment for Insulin Resistance; HRpeak, peak heart rate during a submaximal step test; HRR30, heart rate recovery 30 s after the step test; HRR60, heart rate recovery 60 s after the step test; HRR slope, steepest 30-s slope in heart rate; LPA, light-intensity physical activity; MPA, moderate-intensity physical activity; PA, physical activity; VPA, vigorous-intensity physical activity.

^a^ Postterm group: those born at ≥42 + 0 weeks of gestation.

^b^ Term group (control participants): those born at weeks 39 + 0 to 41 + 6.

^c^ The *P* values indicate 2-sided statistical significance for differences between the postterm and term groups (Student *t* test or Pearson χ^2^ test).

^d^ BMI (kg m^−2^) classified as underweight (BMI < 18.5, healthy weight range (BMI 18.5-24.9), overweight (BMI 25.0-29.9), low-risk obesity (BMI 30.0-34.9), and moderate/high risk obesity (BMI ≥35.0 or ≥40).

^e^ Threshold of 1.9 for HOMA-IR was based on a study^34^ aimed to determine the upper limit of normal HOMA-IR in healthy individuals in 2 Finnish cohorts.

^f^ Self-reported alcohol use classified as nonrisk and at-risk alcohol consumers (above the risk levels of 40 g day^−1^ of pure ethanol for men and 20 g day^−1^ of pure ethanol for women).

^g^ Device-measured daily PA (min day^−1^) determined using a wrist-worn, accelerometer-based monitor and classified as described in the text. Sedentary behavior was assessed as all activity with an intensity of 1-2 metabolic equivalents of task (MET), LPA as all PA with an intensity of 2-3.5 MET, MPA as all activity with an intensity of 3.5-5 MET, and VPA as all activity with an intensity of ≥5 MET.

^h^ Self-reported frequency of leisure PA (causing at least some sweating and panting) classified as high (47 times a week), moderate (1-3 times a week), and low (less than once a week).

^i^ Tertiles according to HRpeak during step tests were formed for men as high (96-138 bpm), moderate (139-153 bpm), and low (154-191 bpm), and for women as high (96-142 bpm), moderate (143-156 bpm), and low (157-192 bpm) to estimate CRF.

### BMI, PA, and muscular strength

Compared with term-born adults, postterm adults had a higher BMI (Table 2). After adjustment for the sex and age at assessment, postterm adults were more likely to have a HOMA-IR value greater than 1.9 (odds ratio [OR] = 1.26; 95% CI, 1.07-1.49) and to be in the group with BMI of at least 35 kg m^−2^ (OR = 1.72; 95% CI, 1.06-2.78), or 30.0-34.9 kg m^−2^ (OR = 1.48; 95% CI, 1.15-1.89) with normal weight (BMI, 18.5-24.9 kg m^−2^) as the reference.

Term-born adults undertook MPA of a mean 38.2 min day^−1^ (SD, 22.2), and VPA of a mean 33.8 min day^−1^ (SD, 21.0) (Table 2). Postterm adults undertook a similar amount of MPA but a mean of 1.94 min day^−1^ less VPA (95% CI, 0.35-3.66) (Figure 3D) than term-born adults when adjusted for the sex and age of assessment (model 1). When model 1 was further adjusted for maternal-, pregnancy-, and childhood-related confounders (model 2), the difference in VPA slightly increased between the postterm and term-born adults. When model 2 was further adjusted for potential adulthood lifestyle and body size–related mediating factors (model 3), the difference in VPA was not statistically significant between the postterm and term-born adults.

**Figure 5:**
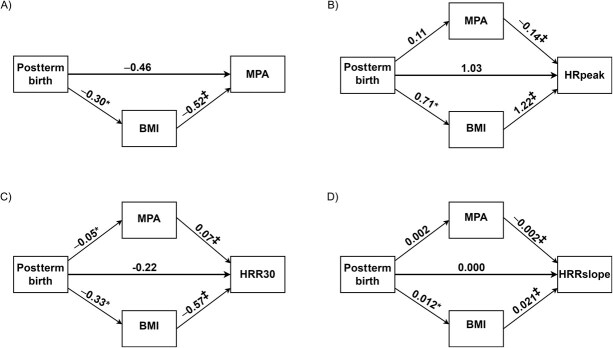
Mediation analysis (Hayes PROCESS, version 4.1.^51^^,^^52^) of the relation between postterm birth with moderate-intensity physical activity (MPA), and heart rate variables among 46-year-old adults from the Northern Finland Birth Cohort 1966. The values are the unstandardized regression coefficients (β) of effects of postterm birth on MPA (min day^−1^) (A), HRpeak (B), HRR30 (C), and HRRslope (D). The mediating variables were MPA and BMI, and the control variables were age and sex at assessment. Estimates are based on a bootstrapping procedure with 10 000 bootstrap samples. ^*^ P < 0.05, and ^‡^  *P* < 0.001 compared with adults born at term. BMI, body mass index; HRpeak, peak heart rate during a submaximal step test; HRR30, heart rate recovery 30 s after the step test; HRRslope, the steepest 30-s slope after the step test; MPA, moderate-intensity physical activity (device-measured).

**Figure 6:**
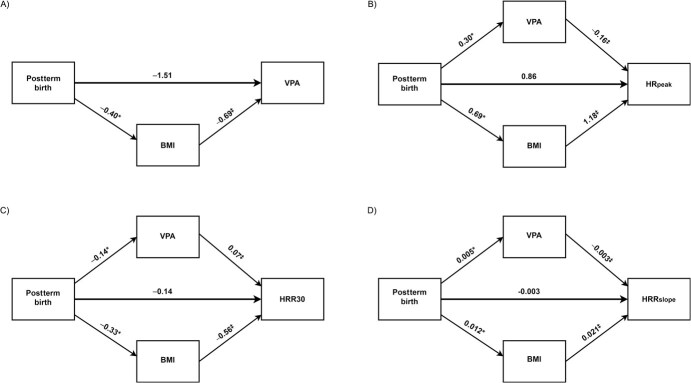
Mediation analysis (Hayes PROCESS, version 4.1.^51^^,^^52^) of the relation between postterm birth with vigorous-intensity physical activity (VPA) and heart rate variables among 46-year-old adults from Northern Finland Birth Cohort 1966. The values are the unstandardized regression coefficients (β) of effects of postterm birth on VPA (min day^−1^) (A), HRpeak (B), HRR30 (C), and HRRslope (D). The mediating variables were VPA and BMI, and the control variables were age and sex at assessment. Estimates are based on a bootstrapping procedure with 10 000 bootstrap samples. ^*^  *P* < 0.05, and ^‡^  *P* < 0.001 compared with adults born at term. BMI, body mass index; HRpeak, peak heart rate during a submaximal step test; HRR30, heart rate recovery 30 sec after the step test; HRRslope, the steepest 30-s slope after the step test; VPA, vigorous-intensity physical activity (device-measured).

We then assessed BMI as a potential mediator of the association between postterm birth and MPA and VPA. Of the total effect (β = −0.77; 95% CI, not significant) of postterm birth on MPA, 39% was mediated through BMI (β = −0.30; 95% CI, −0.50 to −0.13^*^) (Table 3, Figure 5A). Of the total effect of postterm birth on VPA, 79% was a direct effect of postterm birth (β = −1.5; 95% CI, −3.1 to 0.1), and 21% was mediated through BMI (β = −0.4; 95% CI, −0.7 to −0.2^*^) (Table 3, Figure 6A).

**Table 3:** Mediation analysis of the relation between postterm birth with moderate-intensity physical activity, vigorous-intensity physical activity, and heart rate variables.^a^

**Variable** ^b^	**β** ^c^	***P***	**95% CI**	**Variable**	**β** ^c^	***P***	**95% CI**
MPA (min day^−1^)				VPA (min day^−1^)			
Direct effects				Direct effects			
Postterm birth	−0.46	.553	−2.00 to 1.07	Postterm birth	−1.51	.069	−3.13 to 0.12
BMI (kg m^−2^)	−0.52	<.001	−0.68 to −0.35	BMI (kg m^−2^)	−0.69	<.001	−0.86 to −0.51
Total indirect effect^d^				Total indirect effect^d^			
Postterm birth through BMI	−0.30		−0.50 to −0.13^*^	Postterm birth through BMI	−0.40		−0.66 to −0.17^*^
Effects of control variables				Effects of control variables			
Sex	−21.03	<.001	−22.35 to −19.71	Sex	2.38	.009	0.98-3.78
Age, years	0.91	.110	−0.21 to 2.02	Age, years	−0.05	.938	−1.23 to 1.14
Total effect (postterm birth)^e^	−0.77	.328	−2.30 to 0.77	Total effect (postterm birth)^e^	−1.91	.022	−3.55 to −0.27
Peak heart rate, HRpeak, bpm				Peak heart rate, HRpeak, bpm			
Direct effects				Direct effects			
Postterm birth	1.03	.073	−0.10 to 2.16	Postterm birth	0.86	.133	−0.26 to 1.97
MPA (min day^−1^)	−0.14	<.001	−0.16 to −0.11	VPA (min day^−1^)	−0.16	<.001	−0.18 to −0.14
BMI (kg m^−2^)	1.22	<.001	1.10-1.34	BMI (kg m^−2^)	1.18	<.001	1.06-1.31
Total indirect effects^d^	0.82		0.35-1.29^*^	Total indirect effects^d^	0.99		0.52-1.48^*^
Postterm birth through MPA	0.11		−0.10 to 0.31	Postterm birth through VPA	0.30		0.04-0.56^*^
Postterm birth through BMI	0.71		0.32-1.11^*^	Postterm birth through BMI	0.69		0.31-1.07^*^
Effects of control variables				Effects of control variables			
Sex	3.26	<.001	2.16-4.36	Sex	6.54	<.001	5.57-7.50
Age, years	1.48	.004	0.66-2.30	Age, years	1.35	.012	0.53-2.16
Total effect (postterm birth)^e^	1.85	.003	0.64-3.06	Total effect (postterm birth)^e^	1.85	.028	0.64-3.06
HRR30, bpm				HRR30, bpm			
Direct effects				Direct effects			
Postterm birth	−0.22	.437	−0.76 to 0.33	Postterm birth	−0.14	.615	−0.68 to 0.40
MPA (min day^−1^)	0.07	<.001	0.05-0.08	VPA (min day^−1^)	0.07	<.001	0.06-0.08
BMI (kg m^−2^)	−0.57	<.001	−0.63 to −0.51	BMI (kg m^−2^)	−0.56	<.001	−0.62 to −0.50
Total indirect effects^d^	−0.38		−0.61 to −0.16^*^	Total indirect effects^d^	−0.46		−0.69 to −0.24^*^
Postterm birth through MPA	−0.05		−0.15 to −0.05^*^	Postterm birth through VPA	−0.14		−0.26 to −0.02^*^
Postterm birth through BMI	−0.33		−0.52 to −0.15^*^	Postterm birth through BMI	−0.33		−0.51 to −0.15^*^
Effects of control variables				Effects of control variables			
Sex	3.02	<.001	2.48-3.55	Sex	1.46	<.001	0.99-1.93
Age, years	−1.31	<.001	−1.70 to −0.91	Age, years	−1.24	<.001	−1.64 to −0.85
Total effect (postterm birth)^c^	−0.60	.044	−1.18 to −0.02	Total effect (postterm birth)^c^	−0.60	.044	−1.18 to −0.02
HRRslope (beats s^−1^)				HRRslope (beats s^−1^)			
Direct effects				Direct effects			
Postterm birth	0.000	.973	−0.019 to 0.023	Postterm birth	−0.003	.802	−0.022 to 0.017
MPA (min day^−1^)	−0.002	<.001	−0.003 to −0.002	VPA (min day^−1^)	−0.003	<.001	−0.003 to −0.002
BMI (kg m^−2^)	0.021	<.001	0.019-0.023	BMI (kg m^−2^)	0.021	<.001	0.018-0.023
Total indirect effects^d^	0.014		0.006-0.023^*^	Total indirect effects^d^	0.017		0.009-0.026^*^
Postterm birth through MPA	0.002		−0.002 to 0.006	Postterm birth through VPA	0.005		0.001-0.010^*^
Postterm birth through BMI	0.012		0.006-0.019^*^	Postterm birth through BMI	0.012		0.006-0.019^*^
Effects of control variables				Effects of control variables			
Sex	−0.106	<.001	−0.125 to −0.086	Sex	−0.048	<.001	−0.065 to −0.031
Age, years	0.045	<.001	0.031-0.059	Age, years	0.043	<.001	0.029-0.057
Total effect (postterm birth)^e^	0.014	.178	−0.007 to 0.036	Total effect (postterm birth)^e^	0.014	.178	−0.007 to 0.036

Abbreviations: BMI, body mass index; HRpeak, peak heart rate during a submaximal step test; HRR30, heart rate recovery 30 s after the step test; HRRslope, the steepest 30-s slope after the step test; MPA, moderate-intensity physical activity (device-measured); VPA, vigorous-intensity physical activity (device-measured).

^a^ Hayes PROCESS, version 4.1^51^^,^^52^ and mediation model 4 were used for the analysis.

^b^ Mediating variables included MPA, VPA, and BMI; the control variables included age and sex among 46-year-old adults from the Northern Finland Birth Cohort 1966.

^c^ Unstandardized regression coefficient of effects of postterm birth on outcomes.

^d^ Based on bootstrapping with 10 000 bootstrap samples (95% CI).

^e^ Sum of direct and indirect effects.

^*^ *P* < .05.

### CRF and cardiac autonomic function

Among term-born adults, the mean HRpeak during the step test was 147.4 bpm (SD, 15.6). The HRpeak was 1.9 bpm higher (95% CI, 0.7-3.1) in the postterm group than in the term-born group (Figure 4A) when adjusted per model 1. When model 1 was further adjusted per model 2, the difference in HRpeak between the postterm and term-born adults increased. The statistical significance of the difference between the groups remained when model 2 was further adjusted per model 3.

When VPA and BMI were assessed as mediating variables of the association between postterm birth and HRpeak, of the total effect of postterm birth on HRpeak, 46% was a direct effect through postterm birth (β = 0.86; 95% CI, −0.26 to 1.97), 37% was mediated through BMI (β = 0.69; 95% CI, 0.31-1.07^*^), and 16% was mediated through VPA (β = 0.30; 95% CI, 0.04-0.56^*^) (Table 3, Figure 6B). When MPA and BMI were assessed as mediating variables of the association between postterm birth and HRpeak, of the total effect of postterm birth on HRpeak, 56% was a direct effect through postterm birth (β = 1.03; 95% CI, −0.10 to 2.16), 38% was mediated through BMI (β = 0.71; 95% CI, 0.32-1.11^*^), and 6% was mediated through MPA (β = 0.11; 95% CI, −0.10 to 0.31) (Table 3, Figure 5B).

Among term-born adults, the mean HRR30 was 17.6 bpm (SD, 7.6), the mean HRR60 was 28.6 bpm (SD, 8.7), and the mean HRRslope was −0.72 beats s^−1^ (SD, 0.27). The HRR30 was 0.6 bpm (95% CI, 0.02-1.19) less (indicating slower HRR) in the postterm group than in the term-born group when adjusted per model 1 (Figure 4B). The difference between groups for HRR30 remained statistically significant when model 1 was further adjusted per model 2, but the statistical significance of the difference between the groups disappeared when further adjusted per model 3.

When VPA and BMI were assessed as mediating variables of the association between postterm birth and HRR30, of the total effect of postterm birth on HRR30, 23% was a direct effect through postterm birth (β = −0.14; 95% CI, −0.68 to 0.40), 55% was mediated through BMI (β = −0.33; 95% CI, −0.51 to −0.15^*^), and 23% was mediated through VPA (β = −0.14; 95% CI, −0.26 to −0.02^*^) (Table 3, Figure 6C).

The differences between groups on HRV or baroreflex sensitivity variables were not statistically significant (Tables S3 and S4). Sensitivity analyses (when the participants with a gestational age of 45-46 weeks were excluded) showed only a minor effect on the results.

## Discussion

Our main findings were that adults born postterm undertook less vigorous-intensity PA and had reduced CRF compared with those born at term. Adults born postterm may have altered cardiac parasympathetic regulation in middle age. Physical activity and BMI seem to mediate these associations.

High PA is generally associated with good cardiometabolic health^53^ and reduced obesity-related cardiac health risks.^54^ Moderate- to vigorous-intensity PA plays an important role in increasing CRF. It is positively associated with cardiac autonomic regulation^18^^‑^^21^ and longevity,^22^ and inversely associated with all-cause mortality.^13^^‑^^15^^,^^55^ Vigorous-intensity PA is more effective in increasing CRF compared with MPA of equal energy cost.^56^ In addition, VPA has been associated with lower rates of death for cardiovascular health–related reasons compared with MPA,^22^^,^^23^ and a dose response association has been shown for the association of PA level with death.^57^

In our study, postterm adults undertook less VPA than term-born adults. We also found that postterm birth was associated with a higher BMI, which had been observed in recent studies.^1^^,^^9^ Additionally, we showed that an increase in BMI was associated with less PA among postterm adults.

Cardiorespiratory fitness is an important determinant of cardiovascular health in the general population.^13^ High CRF is associated with a lower HR at rest, faster HRR,^20^ and with a lower risk of death associated with obesity.^58^ Low CRF increases the risk of death, regardless of BMI.^15^ A previous study^59^ suggested that slow HRR after exercise was associated with an increased risk of death in healthy, middle-aged men.

Knowledge of CRF and HRR in adults born postterm is incomplete. A previous study among adolescents found that postterm birth was associated with lower exercise capacity, possibly related to changes in the peripheral vascular system and reduced insulin sensitivity.^10^ Also, lower insulin sensitivity among children born postterm has been reported.^11^

In our study, HRpeak was higher and HRR30 slightly slower among adults born postterm than among term-born adults. Although the observed difference in HRR30 was small and may have limited clinical significance, it predicts weaker parasympathetic reactivation and sympathetic withdrawal after exercise later in life.^60^

In our study, a higher BMI and lower PA seemed to mediate the associations of postterm birth with higher HRpeak and slightly lower HRR30. The mediating connection of postterm birth with HRpeak and HRR30 was stronger through VPA than through MPA, although the connection through BMI was even greater. Additionally, adults born postterm had higher HOMA-IR levels than did term-born adults. Explanations for our observations on HRpeak and HRR30 may be lower VPA levels and altered peripheral insulin resistance, like findings in recent studies^10^^,^^11^ regarding younger populations.

Maintenance of a sufficient amount of moderate- to vigorous-intensity PA, including the suitable amount of VPA considering the other health conditions, and weight management are particularly important for adults born postterm. Although a 2-min difference in daily VPA between adults born postterm and at term is rather small, it may not be negligible at a population level, due to the clear dose-dependent association between increasing PA and cardiovascular disease and related death.^61^^‑^^63^ Accordingly, even if we found only small group differences in CRF (corresponding to ~0.15 estimated MET^16^^,^^45^), the difference may have a clinical relevance at population level because 1 MET increase in CRF is associated with greater than 10% decrease in all-cause mortality.^64^

Although postterm births have become less common, due to active delivery induction policies, the genetic factors associated with postterm birth are noteworthy,^65^^,^^66^ and they possibly affect individual lifelong health (eg, metabolic regulation in later life).^67^ Mechanisms underlying the links between postterm birth and lower PA and CRF may include obstetric complications associated with postterm birth or factors regulating parturition. Our findings reinforce previous suggestions that postterm birth should be included as a perinatal risk factor for adult cardiometabolic disease.

Even though our findings did not show clinically significant differences between adults born postterm and term-born adults in terms of baroreflex sensitivity or HRV, the results of HRR may suggest an association between postterm birth and lower vagal cardiac activity with aging, especially in combination with overweight. Future studies should focus on the global impact of postterm birth on health in different populations and even larger data sets using robust methods to access the associations of PA on cardiovascular health. Research on cardiac autonomic function among adults born postterm is also needed.

### Strengths and limitations

Strengths of this study include comprehensive PA, fitness, and cardiac autonomic function measurements, and sufficient power to detect all but very small differences (an effect size of 0.11 SD units) between the term and postterm groups. A limitation was that gestational age was based on the maternally reported last menstrual period, because ultrasound was unavailable at that time. This may have resulted in increased random errors and less precise estimates. Regarding CRF determination, we could not exclude the possibility of bias caused by individual differences in HRpeak nor that due to the submaximal step test method. However, this method has been validated, and it is widely accepted.^68^ Physical activity was measured with a wrist-worn accelerometer, and the lack of posture recognition is a limitation, because the physiological consequences of sitting and standing can be different. Also, the intensity of specific activities, such as cycling or carrying loads, might be underestimated. Moreover, the self-report questionnaire was completed at a different time from accelerometer use and thus did not measure the same period. The study population did not fully represent the entire cohort, due to incomplete participation in the clinical examinations at 46 years of age (57% of the invited individuals). Although postterm birth was unrelated to participation, study participants were more likely to have been sports club members as adolescents than nonparticipants, especially among term-born adults, thus possibly representing the more physically active and physically fit part of the cohort. Moreover, the study population was reduced due to the exclusion of individuals who were using β-blockers or those who had missing outcome variables. Finally, residual confounding by unmeasured environmental and lifestyle factors could not be excluded.

In conclusion, adults born postterm undertook less vigorous-intensity PA and had reduced CRF compared with those born at term, and they may have altered cardiac parasympathetic regulation in middle age. Lifestyle and environmental or genetic factors may modify these associations. Although the mechanisms remain uncertain, our findings reinforce previous suggestions that postterm birth should be included as a perinatal risk factor for adult cardiometabolic disease.

## Acknowledgments

We thank all cohort members and researchers who participated in the 46-year study. We also wish to acknowledge the work of the NFBC Project Center (University of Oulu, Finland). Finally, we thank statistician Markku Nurhonen (Finnish Institute for Health and Welfare, Helsinki, Finland) for his guidance regarding statistical methods. This work was presented in part at the Puijo Symposium, Kuopio, Finland, on June 28, 2022; and at the Liikuntalääketieteen päivät (sports medicine conference), Helsinki, Finland, in November 2023.

## Supplementary Material

Web_Material_kwae150

## Data Availability

All data were used in accordance with the European Union General Data Protection Regulation (679/2016) and the Finnish Data Protection Act. NFBC data are available from the University of Oulu, Infrastructure for Population Studies. The data are not publicly available due to privacy or ethical restrictions. Permission to use the data for research purposes can be requested via an electronic request portal. The cohort participants provided written informed consent during the latest follow-up study, which may place limitations on the use of their personal data. For more information, please contact the NFBC project center (NFBCprojectcenter@oulu.fi) or visit the cohort website (www.oulu.fi/nfbc).
