# Supplementary material for: Physical activity, fitness, and cardiac autonomic function among adults born postterm
Source: Am J Epidemiol. 2024 Jun 24;194(3):766–78. doi: 10.1093/aje/kwae150 (PMC11879586; doi:10.1093/aje/kwae150)
Supplement: Web_Material_kwae150 [file web_material_kwae150.docx]

**Supplementary Data**

**Physical Activity, Fitness, and Cardiac Autonomic Function among Adults Born Postterm**

Päivi Oksanen*, Marjaana Tikanmäki, Mikko P Tulppo, Maisa Niemelä, Raija Korpelainen, and Eero Kajantie

**Contents:**

Table S1

Table S2

Appendix S1

Table S3

Table S4

**Table S1.** Variables in Multiple Regression Models 1–3 among Adults Born Postterm and at Term from Northern Finland Birth Cohort 1966

|  | **Model 1** | **Model 2** | **Model 3** | **No. missing** |
| --- | --- | --- | --- | --- |
| Age (years) | ✔ | ✔ | ✔ | 0 |
| Sex | ✔ | ✔ | ✔ | 0 |
| **Maternal- and pregnancy-related variables** |  |  |  |  |
| Maternal body mass index^a^ (kg/m^2^) before pregnancy |  | ✔ | ✔ | 236 |
| Maternal smoking in the second month of pregnancy |  | ✔ | ✔ | 65 |
| Maternal diabetes mellitus^b^ (manifest or latent: prediabetes) |  | ✔ | ✔ | 88 |
| Maternal hypertension in 2nd–4th and 5th–9th months (gestational^c^ or chronic^d^) |  | ✔ | ✔ | 73 |
| Maternal preeclampsia^e^ or superimposed preeclampsia^f^ |  | ✔ | ✔ |  |
| Maternal age at delivery (years) |  | ✔ | ✔ | 0 |
| Parity (at least 1 partus before this pregnancy) |  | ✔ | ✔ | 0 |
| Multiple deliveries |  | ✔ | ✔ | 0 |
| Birth weight standard deviation (*SD*) scores |  | ✔ | ✔ | 5 |
| Self-reported maternal occupation^g^ |  | ✔ | ✔ | 0 |
| Self-reported maternal occupation^g^ |  | ✔ | ✔ | 0 |
| **Childhood-related variable (at 14 years of age)** |  |  |  |  |
| Paternal smoking (reported by cohort participant or their parents) |  | ✔ | ✔ | 250 |
| **Adulthood lifestyle- and body size-related factors** |  |  |  |  |
| Body mass index (kg/m^2^) |  |  | ✔ | 0 |
| Height (cm) |  |  | ✔ | 0 |
| Current smoking |  |  | ✔ | 150 |
| Alcohol use (g/day)^h^ |  |  | ✔ | 131 |

^a^ Maternal body mass index (BMI) in early pregnancy (based on maternal self-reported weight and height before pregnancy or measured values during first visit at maternal welfare clinic) classified as healthy weight range (BMI < 25.0 kg/m^2^), overweight (BMI 25.0 to 29.9 kg/m^2^), obesity (BMI ≥ 30.0 kg/m^2^).

^b^ Glycemic control was assessed on the basis of a urine sample.

^c^ Gestational hypertension: blood pressure 145/95 mmHg or more after 20th gestational week. Normal blood pressure in the early pregnancy (<20th gestational week). No proteinuria.

^d^ Chronic hypertension: a. blood pressure elevated already in early pregnancy (<20th gestational week), 145/95 mmHg or more, and blood pressure elevated also during whole pregnancy or after pregnancy (6weeks after pregnancy) b. Chronic hypertension (hypertension essentialis) diagnose registered in questionnaire.

^e^ Pre-eclampsia: blood pressure 145/95 mmHg or more after 20th gestational week. Normal blood pressure in the early pregnancy (<20th gestational week). Proteinuria at least in one sample during pregnancy.

^f^ Superimposed pre-eclampsia: Chronic hypertension and proteinuria.

^g^ Professional, Skilled or unskilled manual worker, No occupation.

^h^ Self-reported alcohol use classified as non-risk and at-risk alcohol consumers (above the risk levels of 40 g/day of pure ethanol for men and 20 g/day of pure ethanol for women).

Of 2,645 adults born at term, 2,119 had complete data regarding outcomes and predicting variables.

Of 805 adults born postterm, 630 had complete data regarding outcomes and predicting variables.

**Table S2.** Perinatal (mother and child), Neonatal and Childhood Characteristics among Northern Finland Birth Cohort 1966 Members Included in the Present Study and Cohort Members Who Did Not Have Sufficient Data for the Study. Separate Comparisons for Those Born Postterm and the Comparison Group of Those Born at Term

| **Variable** | **Postterm^a^ (*n* = 2203)** | | | | |  | **Term^b^ (*n* = 7379)** | | | | |
| --- | --- | --- | --- | --- | --- | --- | --- | --- | --- | --- | --- |
|  | **Participants (*n* = 805)** | | **Cohort members with insufficient data (*n* = 1,398)** | |  |  | **Participants (*n* = 2,645)** | | **Cohort members with insufficient data (*n* = 4,734)** | |  |
|  | **Mean (*SD*)/ Median [IQR]** | **No. (%)** | **Mean (*SD*)/ Median [IQR]** | **No. (%)** | ***P*^c^** |  | **Mean (*SD*)/ Median [IQR]** | **No. (%)** | **Mean (*SD*)/ Median [IQR]** | **No. (%)** | ***P*^c^** |
| Participants |  | 805 (36.5) |  |  |  |  |  | 2,645 (35.8) |  |  |  |
| Male |  | 385 (47.8) |  | 718 (51.4) | 0.110 |  |  | 1,189 (45.0) |  | 2,590 (54.7) | <0.001 |
| Peri- and neonatal |  |  |  |  |  |  |  |  |  |  |  |
| Maternal age at delivery | 27.4 (6.7) |  | 27.3 (7.2) | 0.557 |  |  | 27.8 (6.9) |  | 27.4 (7.1) |  | 0.021 |
| Maternal smoking^d^ |  | 94 (11.7) |  | 229 (16.4) | 0.003 |  |  | 309 (11.7) |  | 772 (16.3) | <0.001 |
| Maternal diabetes^e^ |  | 2 (0.2) |  | 0 (0.0) | 0.133* |  |  | 0 (0.0) |  | 2 (0.0) | 0.540* |
| Maternal hypertension^f^ |  | 109 (13.5) |  | 240 (17.2) | 0.025 |  |  | 326 (12.3) |  | 638 (13.5) | 0.159 |
| Maternal preeclampsia^g^ |  | 22 (2.7) |  | 45 (3.2) | 0.522 |  |  | 66 (2.5) |  | 122 (2.6) | 0.831 |
| Maternal BMI^h^ (kg/m^2^) | 23.0 (3.1) |  | 23.3 (3.4) |  | 0.049 |  | 23.1 (3.1) |  | 23.1 (3.3) |  | 0.554 |
| BMI < 25.0 |  | 595 (73.9) |  | 952 (68.1) | 0.004 |  |  | 1,949 (73.7) |  | 3,351 (70.8) |  |
| BMI 25.0 to 29.9 |  | 122 (15.2) |  | 258 (18.5) | 0.048 |  |  | 442 (16.7) |  | 810 (17.1) |  |
| BMI ≥ 30.0 |  | 26 (3.2) |  | 53 (3.8) | 0.495 |  |  | 80 (3.0) |  | 181 (3.8) |  |
| Multiple deliveries |  | 3 (0.4) |  | 7 (0.5) | 0.755* |  |  | 33 (1.2) |  | 70 (1.5) | 0.417 |
| Parity | 2.80 [3.00] |  | 2.89 [3.00] |  | 0.749** |  | 2.86 [3.00] |  | 2.86 [3.00] |  | 0.920** |
| Birth weight *SD* score | 0.11 (0.98) |  | 0.00 (1.05) | 0.011 |  |  | –0.14 (1.00) |  | –0.24 (1.0) |  | <0.001 |
| Small for gestational age |  | 15 (1.9) |  | 34 (2.4) | 0.383 |  |  | 64 (2.4) |  | 195 (4.1) | <0.001 |
| Large for gestational age |  | 24 (3.0) |  | 44 (3.1) | 0.828 |  |  | 59 (2.2) |  | 80 (1.7) | 0.101 |
| Maternal occupation |  |  |  |  | 0.130 |  |  |  |  |  | 0.003 |
| Professional |  | 126 (15.7) |  | 176 (12.6) |  |  |  | 387 (14.6) |  | 570 (12.0) |  |
| Manual worker^i^ |  | 243 (30.2) |  | 433 (31.0) |  |  |  | 776 (29.3) |  | 1,368 (28.9) |  |
| No occupation or not known |  | 436 (54.2) |  | 789 (56.4) |  |  |  | 1,482 (56.0) |  | 2,796 (59.1) |  |
| Paternal occupation |  |  |  |  | 0.049 |  |  |  |  |  | <0.001 |
| Professional |  | 212 (26.3) |  | 317 (22.7) |  |  |  | 696 (26.3) |  | 1,053 (22.2) |  |
| Manual worker^i^ |  | 411 (51.1) |  | 787 (56.3) |  |  |  | 1,301 (49.2) |  | 2,638 (55.7) |  |
| No occupation or not known |  | 182 (22.6) |  | 294 (21.0) |  |  |  | 648 (24.5) |  | 1,043 (22.0) |  |
| At around 14 years |  |  |  |  |  |  |  |  |  |  |  |
| Paternal smoking |  | 225 (28.0) |  | 489 (35.0) | 0.001 |  |  | 923 (34.9) |  | 1,660 (35.1) | 0.884 |
| Membership in a sports club |  | 290 (37.7) |  | 437 (35.8) | 0.394 |  |  | 974 (38.3) |  | 1,447 (34.8) | 0.004 |

Abbreviations: BMI, body mass index; No., number; *SD*, standard deviation.

^a^ Postterm group: those born at ≥42+0 weeks of gestation.

^b^ Term group (controls): those born at weeks 39+0–41+6.

^c^ The *P* values concern two-sided statistical significance for differences between the participants and cohort members with insufficient data using Pearson’s χ2 test (No. (%)), Fisher's Exact Test* (No. (%)), Student’s t-test (Mean (*SD*)) or Mann-Whitney U-test** (Median [IQR, Interquartile range]).

^d^ Maternal smoking during pregnancy.

^e^ Diabetes mellitus or prediabetes.

^f^ Gestational or chronic hypertension.

^g^ Preeclampsia or superimposed preeclampsia.

^h^ BMI in early pregnancy classified as healthy weight range (BMI <25.0 kg/m^2^), overweight (BMI 25.0 to 29.9 kg/m^2^), obesity (BMI ≥30.0 kg/m^2^).

^i^ Skilled or unskilled manual worker

**Appendix S1**

**SPSS Code for Dummy Variables**

Categorical covariates were entered to analyses as dummy variables due to their non-numeric nature. An example (a variable for maternal hypertension and preeclampsia) of SPSS code creation for dummy variables is shown below.

*C6600_UURRHYLK= mat_hypertensio_preecl

*Maternal hypertension or preeclampsia in pregnancy. momhypertens-variable dummycoded, missings are coded as a separate variable:

recode C6600_UURRHYLK (1,3=1)(2,4,5,6,7,8,9,sysmis=0) into GHTdum1.

variable labels GHTdum1 'GHTdum1, maternal gestational or chronic hypertension, dummycoded from mat_ hypertensio_preecl'.

value labels GHTdum1 1 '1,maternal GHT or CHT' 0 '0, normotensive, SBP elevated, DBP elevated, PE, superimp PE, Could not be determined, missing'.

formats GHTdum1(f1).

recode C6600_UURRHYLK (2,4=1)(1,3,5,6,7,8,9,sysmis=0) into GHTdum2.

variable labels GHTdum2 'GHTdum2, maternal preeclampsia or superimposed PE, dummycoded from mat_ hypertensio_preecl'.

value labels GHTdum2 1 '1,maternal PE or superimposed PE' 0 '0, normotensive, SBP elevated, DBP elevated, GHT or CHT, missing'.

formats GHTdum2(f1).

recode C6600_UURRHYLK (sysmis=1)(1,2,3,4,5,6,7,8,9=0) into GHTdum3.

variable labels GHTdum3 'GHTdum3, missing maternal hypertension in pregnancy, dummycoded from mat_ hypertensio_preecl'.

value labels GHTdum3 1 '1,missing' 0 '0, normotensive, SBP elevated, DBP elevated ,GHT or CHT, PE or superimposed PE'.

formats GHTdum3(f1).

**Table S3.** Heart Rate Variability during an Orthostatic Test^a^ among 46-Year-Old Adults Born Term and Postterm From Northern Finland Birth Cohort 1966

| **Outcome** |  | **Term^b^ (*n* = 2,645)** |  | **Postterm^c^ (*n* = 805)** | **Mean difference (95% CI) between groups** | **No. missing Term/Postterm** |
| --- | --- | --- | --- | --- | --- | --- |
|  | **Model^d^** | **Mean (*SD*)** |  | **Mean (*SD*)** |  |  |
| RRi, seated (ms) |  | 860.6 (131.6) |  | 858.8 (131.3) | −1.8 (−12.1, 8.6) |  |
|  | 1 |  |  |  | −2.8 (−13.2, 7.5) |  |
|  | 2 |  |  |  | −3.6 (−14.0, 6.9) |  |
|  | 3 |  |  |  | 1.4 (−8.9, 11.6) |  |
| RRi, standing (ms) |  | 748.1 (115.5) |  | 748.5 (117.6) | 0.4 (−8.6, 9.6) | 2/1 |
|  | 1 |  |  |  | −0.7 (−9.8, 8.5) |  |
|  | 2 |  |  |  | −0.7 (−9.9, 8.5) |  |
|  | 3 |  |  |  | 1.3 (−7.9, 10.5) |  |
| rMSSD, seated (ms) |  | 26.5 (16.3) |  | 26.6 (17.1) | 0.04 (−1.25, 1.34) |  |
|  | 1 |  |  |  | 0.04 (−1.25, 1.33) |  |
|  | 2 |  |  |  | 0.04 (−1.27, 1.34) |  |
|  | 3 |  |  |  | 0.40 (−0.90, 1.69) |  |
| rMSSD, standing (ms) |  | 15.5 (10.1) |  | 15.6 (10.7) | 0.12 (−0.69, 0.93) |  |
|  | 1 |  |  |  | 0.01 (−0.80, 0.82) |  |
|  | 2 |  |  |  | 0.01 (−0.81, 0.82) |  |
|  | 3 |  |  |  | 0.06 (−0.75, 0.88) |  |
| LFP, seated (ln ms^2^) |  | 5.84 (0.97) |  | 5.85 (0.98) | 0.01 (−0.06, 0.09) |  |
|  | 1 |  |  |  | 0.01 (−0.07, 0.08) |  |
|  | 2 |  |  |  | 0.00 (−0.07, 0.08) |  |
|  | 3 |  |  |  | 0.03 (−0.05, 0.11) |  |
| LFP, standing (ln ms^2^) |  | 5.52 (0.99) |  | 5.49 (1.00) | −0.02 (−0.10, 0.06) |  |
|  | 1 |  |  |  | −0.04 (−0.11, 0.04) |  |
|  | 2 |  |  |  | −0.04 (−0.12, 0.04) |  |
|  | 3 |  |  |  | −0.02 (−0.10, 0.06) |  |
| HFP, seated (ln ms^2^) |  | 5.35 (1.24) |  | 5.31 (1.24) | −0.04 (−0.14, 0.06) |  |
|  | 1 |  |  |  | −0.04 (−0.13, 0.06) |  |
|  | 2 |  |  |  | −0.04 (−0.14, 0.06) |  |
|  | 3 |  |  |  | −0.01 (−0.10, 0.09) |  |
| HFP, standing (ln ms^2^) |  | 4.33 (1.21) |  | 4.25 (1.27) | −0.08 (−0.18, 0.02) |  |
|  | 1 |  |  |  | −0.08 (−0.18, 0.02) |  |
|  | 2 |  |  |  | −0.08 (−0.18, 0.01) |  |
|  | 3 |  |  |  | −0.07 (−0.17, 0.02) |  |
| LFP/HFP ratio, seated |  | 2.52 (2.74) |  | 2.64 (3.18) | 0.12 (−0.11, 0.34) |  |
|  | 1 |  |  |  | 0.09 (−0.12, 0.31) |  |
|  | 2 |  |  |  | 0.09 (−0.13, 0.31) |  |
|  | 3 |  |  |  | 0.08 (−0.14, 0.30) |  |
| LFP/HFP ratio, standing |  | 4.76 (4.71) |  | 4.98 (4.77) | 0.23 (−0.15, 0.60) | 0/1 |
|  | 1 |  |  |  | 0.18 (−0.19, 0.54) |  |
|  | 2 |  |  |  | 0.14 (−0.22, 0.51) |  |
|  | 3 |  |  |  | 0.17 (−0.19, 0.54) |  |
| DFAα1, seated |  | 1.15 (0.28) |  | 1.16 (0.27) | 0.02 (−0.00, 0.04) |  |
|  | 1 |  |  |  | 0.02 (−0.00, 0.04) |  |
|  | 2 |  |  |  | 0.02 (−0.00, 0.04) |  |
|  | 3 |  |  |  | 0.02 (−0.00, 0.04) |  |
| DFAα1, standing |  | 1.38 (0.25) |  | 1.38 (0.24) | −0.00 (−0.02, 0.02) |  |
|  | 1 |  |  |  | −0.00 (−0.02, 0.02) |  |
|  | 2 |  |  |  | −0.00 (−0.02, 0.02) |  |
|  | 3 |  |  |  | −0.00 (−0.02, 0.02) |  |

Abbreviations: BMI, body mass index; CI, confidence interval; DFAα1, short-term scaling exponent of RRi detrended fluctuation analysis; HFP, high-frequency power of heart rate variability; LFP, low-frequency power of heart rate variability; No., number; rMSSD, root mean square of successive differences between normal heart beats; RRi, R–R interval (the time elapsed between two successive R-waves of the QRS signal); *SD*, standard deviation.

^a^ Orthostatic test: 3 min seated and 3 min standing.

^b^ Term group (controls): those born at weeks 39+0–41+6.

^c^ Postterm group: those born at ≥42+0 weeks of gestation.

^d^ Multiple linear regression analyses were adjusted for three models. Model 1 included sex and age at assessment. Model 2 included the Model 1 variables and as additional confounders maternal-, pregnancy-, and childhood-related factors (maternal BMI (kg/m^2^) before pregnancy, maternal smoking in the second month of pregnancy, maternal diabetes, maternal hypertension, maternal preeclampsia, parity, maternal age at delivery, multiple deliveries, birth weight *SD* score, self-reported parental occupation, and paternal smoking during childhood). Model 3 included Model 2 variables and adulthood lifestyle-related covariates (smoking, alcohol consumption over the risk level, BMI (kg/m^2^), and height).

**Table S4.** Baroreflex Sensitivity during an Orthostatic Test^a^ among 46-Year-Old Adults Born Term and Postterm from Northern Finland Birth Cohort 1966

| **Outcome** |  | **Term^b^ (*n* = 1280)** |  | **Postterm^c^ (*n* =368)** | **Mean difference (95% CI)  between groups** | **No. missing Term/Postterm** |
| --- | --- | --- | --- | --- | --- | --- |
|  | **Model^d^** | **Mean (*SD*)** |  | **Mean (*SD*)** |  |  |
| SBP, seated (mmHg) |  | 118.6 (15.7) |  | 119.4 (16.0) | 0.8 (−1.0, 2.6) |  |
|  | 1 |  |  |  | 0.7 (−1.0, 2.5) |  |
|  | 2 |  |  |  | 0.8 (−1.0, 2.6) |  |
|  | 3 |  |  |  | 0.5 (−1.2, 2.3) |  |
| SBP, standing (mmHg) |  | 116.9 (15.6) |  | 117.2 (15.4) | 0.3 (−1.5, 2.1) | 8/1 |
|  | 1 |  |  |  | 0.3 (−1.5, 2.0) |  |
|  | 2 |  |  |  | 0.3 (−1.5, 2.1) |  |
|  | 3 |  |  |  | −0.1(−1.8, 1.7) |  |
| DBP, seated (mmHg) |  | 71.1 (8.8) |  | 71.4 (9.1) | 0.3 (−0.8, 1.3) |  |
|  | 1 |  |  |  | 0.3 (−0.7, 1.3) |  |
|  | 2 |  |  |  | 0.3 (−0.7, 1.3) |  |
|  | 3 |  |  |  | 0.2 (−0.8, 1.2) |  |
| DBP, standing (mmHg) |  | 73.2 (8.9) |  | 73.3 (8.8) | 0.1 (−0.9, 1.1) | 8/1 |
|  | 1 |  |  |  | 0.1 (−0.9, 1.6) |  |
|  | 2 |  |  |  | 0.2 (−0.8, 1.2) |  |
|  | 3 |  |  |  | 0.0 (−1.0, 1.0) |  |
| LFSBPV, seated (ms^2^) |  | 7.87 (8.00) |  | 7.85 (7.72) | –0.02 ( −0.94, 0.90) |  |
|  | 1 |  |  |  | 0.04 (−0.89, 0.96) |  |
|  | 2 |  |  |  | 0.01 (−0.93, 0.94) |  |
|  | 3 |  |  |  | −0.00 (−0.94, 0.93) |  |
| LFSBPV, standing (ms^2^) |  | 12.02 (11.83) |  | 11.45 (12.60) | −0.57 (−1.97, 0.83) | 8/1 |
|  | 1 |  |  |  | −0.60 (−2.00, 0.80) |  |
|  | 2 |  |  |  | −0.85 (−2.26, 0.57) |  |
|  | 3 |  |  |  | −0.91 (−2.32, 0.50) |  |
| α-LF, seated (ms/mmHg) |  | 7.55 (3.98) |  | 7.48 (4.08) | −0.08 (−0.54, 0.39) |  |
|  | 1 |  |  |  | −0.18 (−0.64, 0.28) |  |
|  | 2 |  |  |  | −0.20 (−0.66, 0.26) |  |
|  | 3 |  |  |  | −0.12 (−0.57, 0.34) |  |
| α-LF, standing (ms/mmHg) |  | 5.22 (2.84) |  | 5.41 (3.43) | 0.19 (−0.15, 0.54) | 11/1 |
|  | 1 |  |  |  | 0.09 (−0.25, 0.43) |  |
|  | 2 |  |  |  | 0.13 (−0.21, 0.47) |  |
|  | 3 |  |  |  | 0.20 (−0.13, 0.53) |  |

Abbreviations: BMI, body mass index; CI, confidence interval; DBP, diastolic blood pressure; LFSBPV, low-frequency SBP variability; No., number; SBP, systolic blood pressure; α-LF, low-frequency band as the square root of the R–R interval/SBP spectral component ratio; *SD*, standard deviation.

^a^ Orthostatic test: 3 min seated and 3 min standing.

^b^ Term group (controls): those born at weeks 39+0–41+6.

^c^ Postterm group: those born at ≥42+0 weeks of gestation.

^d^ Multiple linear regression analyses were adjusted for three models. Model 1 included sex and age at assessment. Model 2 included the Model 1 variables and as additional confounders maternal-, pregnancy-, and childhood-related factors (maternal BMI (kg/m^2^) before pregnancy, maternal smoking in the second month of pregnancy, maternal diabetes, maternal hypertension, maternal preeclampsia, parity, maternal age at delivery, multiple deliveries, birth weight *SD* score, self-reported parental occupation, and paternal smoking during childhood). Model 3 included Model 2 variables and adulthood lifestyle-related covariates (smoking, alcohol consumption over the risk level, BMI (kg/m^2^), and height).
